# Application of Data-Independent Acquisition Approach to Study the Proteome Change from Early to Later Phases of Tomato Pathogenesis Responses

**DOI:** 10.3390/ijms20040863

**Published:** 2019-02-17

**Authors:** Kai-Ting Fan, Kuo-Hsin Wang, Wei-Hung Chang, Jhih-Ci Yang, Ching-Fang Yeh, Kai-Tan Cheng, Sheng-Chi Hung, Yet-Ran Chen

**Affiliations:** 1 Agricultural Biotechnology Research Center, Academia Sinica, Taipei 11529, Taiwan; kaitingfan@sinica.edu.tw (K.-T.F.); khwang@gate.sinica.edu.tw (K.-H.W.); whchang@gate.sinica.edu.tw (W.-H.C.); cfyeh@gate.sinica.edu.tw (C.-F.Y.); ktc77123@gate.sinica.edu.tw (K.-T.C.); 2 Sustainable Chemical Science and Technology, Taiwan International Graduate Program, Institute of Chemistry, Academia Sinica, Taipei 11529, Taiwan; jhihciy@gate.sinica.edu.tw; 3 Sustainable Chemical Science and Technology, Taiwan International Graduate Program, Department of Applied Chemistry, National Chiao Tung University, Hsinchu 30010, Taiwan; kempis710165@gate.sinica.edu.tw; 4 Institute of Biotechnology, National Taiwan University, Taipei 10617, Taiwan

**Keywords:** plant pathogenesis responses, data-independent acquisition, quantitative proteomics, *Pseudomonas syringae*

## Abstract

Plants and pathogens are entangled in a continual arms race. Plants have evolved dynamic defence and immune mechanisms to resist infection and enhance immunity for second wave attacks from the same or different types of pathogenic species. In addition to evolutionarily and physiological changes, plant-pathogen interaction is also highly dynamic at the molecular level. Recently, an emerging quantitative mass spectrometry-based proteomics approach named data-independent acquisition (DIA), has been developed for the analysis of the proteome in a high-throughput fashion. In this study, the DIA approach was applied to quantitatively trace the change in the plant proteome from the early to the later stage of pathogenesis progression. This study revealed that at the early stage of the pathogenesis response, proteins directly related to the chaperon were regulated for the defence proteins. At the later stage, not only the defence proteins but also a set of the pathogen-associated molecular pattern-triggered immunity (PTI) and effector triggered immunity (ETI)-related proteins were highly induced. Our findings show the dynamics of the plant regulation of pathogenesis at the protein level and demonstrate the potential of using the DIA approach for tracing the dynamics of the plant proteome during pathogenesis responses.

## 1. Introduction

Plants are sessile organisms that are in close contact with a variety of organisms, including pathogens and have thus evolved an efficient innate immune system to defend themselves from those biotic stresses. The typical passive defence of plants actually starts from physical barriers, including the trichomes, waxy cuticle and cell wall [1]. On the other hand, successful active defence responses are initiated from the ability of plants to sense pathogens using extracellular and intracellular innate immune receptors, to induce subsequent cellular reprogramming for defence. Plants have evolved to express receptors that recognize conserved pathogen-associated molecular patterns (PAMPs) or microbial associated molecular patterns (MAMPs) such as FLS2 receptor that perceives bacterial flagellin via the minimal epitope flg22, one of conserved pathogen molecules essential for its reproduction [2]. Another type of plant sensing ability is initiated by endogenous damaged-associated molecular patterns (DAMPs) which are molecules produced by cell-damage or necrosis caused by pathogen invasion or herbivore attack, including fragments of cell wall structure, signalling peptides/polypeptides from cleaved precursor proteins like systemin [3], PLANT ELICITOR PEPTIDES (PEPs) [4] and CAP-derived peptide 1 (CAPE1) [5] and extracellular molecules like nucleotides [6]. Currently known DAMPs have been demonstrated to be able to induce similar innate immune responses in plants as microbe-derived PAMPs/MAMPs and it has been proposed that DAMPs could be used to amplify the responses triggered by PAMPs [7].

After perceiving the danger signals of pathogen attacks, plants initiate a series of defence responses. The first one, often thought as the basal pathogen resistance of plants, is triggered by the binding of PAMPs/MAMPs by the plasma membrane-localized receptor (pattern recognition receptors; PRRs) and is therefore called PAMP-triggered immunity (PTI) [8]. PTI includes the increased ion influx, a burst of reactive oxygen species, activation of the mitogen-activated protein kinase (MAPK) cascade and increased level of defence phytohormones like salicylic acid (SA), jasmonic acid (JA) or ethylene (ET). However, pathogens that have become successfully adapted though evolution can suppress or bypass the PTI responses by injecting proteins—termed effectors through a type III secretion system to the apoplast or cellular region of the host, resulting in effector-triggered susceptibility (ETS) [9,10]. To counter-attack ETS, plant species have also evolved another kind of defence response, effector-triggered immunity (ETI), which uses intracellular or transmembrane receptors (R proteins) to specifically target the effector proteins of pathogens, resulting in much stronger resistance responses, usually leading to hypersensitive response (HR) cell death. A “zig-zag” model has been proposed to interpret how/why the plant immune system is made up of this complex, multi-layered innate immune system of PTI and ETI responses through evolutionary development [10]. Although from the transcriptomics data, a significant number of genes could both be regulated by PTI and ETI, the later causes faster and greater amplitude of induction, leading scientists to speculate that PTI and ETI could result in synergistic effects [11,12,13]. However, when examining specific defence-related phytohormones (SA, JA and ET) in Arabidopsis, it has been shown that in PTI there are more evident synergistic relationships; while in ETI there are more compensatory relationships among the signalling sectors [14,15,16]. There are still gaps in understanding how these sophisticated mechanisms are regulated such as how PTI and ETI together form an effective defence network, since a large number of the transcripts involved in the regulation of immune and defence response are known to be time-dependent [17]. This is also related to the fact that regulation at the protein level should also be time dependent and highly dynamic.

The isobaric labelling approach has been used to study the dynamics of proteome regulation in plants during pathogenesis responses. This approach has been used to trace the change in the proteome of tomato in response to infection with *Pseudomonas syringae* pv. *tomato* (*Pst*) DC3000 at early and late time points. Using a combination of strong off-line cation exchange chromatography and liquid chromatography coupled with mass spectrometry (LC-MS), a total of ~2300 proteins were identified [18]. Although the isobaric approach is a promising technology to perform multiplex quantitative proteomics analysis, it is often required to fractionate the sample to minimize the effect of ratio compression [19]. The use of an additional fractionation step will require a higher quantity of sample to compensate for sample loss during the fractionation step and reduce the throughput of the proteome analysis. This limits its application to the analysis of a large quantity of the proteomics samples. Recently, a new MS analysis approach called data independent acquisition (DIA) was proposed to solve this issue. The DIA approach is considered a promising approach for performing quantitative proteome profiling in a high-throughput manner. The DIA approach is based on the acquisition of fragment-ion information for all precursor ions within a certain range of m/z values, as demonstrated by the sequential window acquisition of all theoretical mass spectra (SWATH) approach [20]. It has been demonstrated that the application of the DIA approach was able to identify and quantify thousands of proteins without performing fractionation and only a few micrograms of the protein sample is required [20]. In the DIA approach, selected reaction monitoring (SRM)-like extracted ion chromatography (XIC) on sequence specific ion transitions can be used for the identification and SRM-like quantification of the peptides after the data acquisition. To demonstrate this technology in the study of the plant pathogenesis responses, here we used the tomato proteome regulated by infection of *Pst* DC3000. *P. syringae*, a hemi-biotroph bacteria, is one of the most studied bacterial pathogens due to its ability to infect a great variety of plant species including reference plants like Arabidopsis and tobacco and crops like tomato and potato. *P. syringae* relies on effector proteins belonging to the bacterial type III secretion system to suppress the plant defence system thus achieving pathogenesis. More than 40 *P. syringae* effectors have been identified with their host targets in plants, demonstrating complex plant-pathogen interactions which makes *P. syringae* an important model system for examining the molecular mechanisms of plant pathogen defence [21]. We quantitatively profiled the total tomato proteomes regulated by the infection of *Pst* DC3000 from early to later time points using the LC-MS/MS operated in DIA mode. Using this approach, the change in the proteomes of mock and *Pst* DC3000-inoculated samples were analysed to identify the proteins with significant change in abundance in the early to the later stage of the pathogenesis responses. Without the hideous labour requirement for sample preparation that is required for peptide fractionation, the one-shot sample analysis using the DIA approach should provide researchers an efficient way to identify not only well-known but also potential protein markers for further biological studies.

## 2. Results and Discussion

### 2.1. Experimental Design and the Identification and Quantification Result

**Figure 1:**
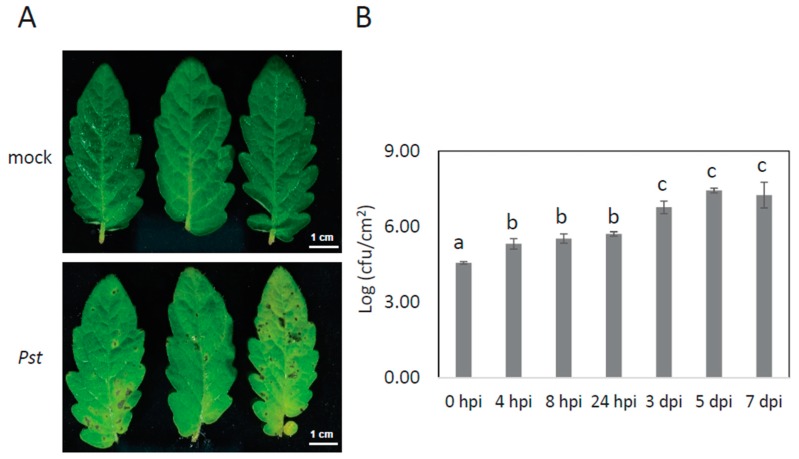
Disease phenotypes and population dynamics of *Pst* DC3000 on tomato leaves. (**A**) Visible disease symptoms in tomato leaves after leaves were dipped into bacterial suspension at an OD_600_ of 0.02. Photos were taken at 5 days post-inoculation. (**B**) Bacteria titres in leaves at 0, 4, 8, 24 h or 3, 5, 7 days after inoculation with *Pst* DC3000. Error bars represent the standard deviation of three biological replicates. A statistical significance difference is shown between different time points using analysis of variance (ANOVA) with post-hoc Tukey’s honestly significant difference (HSD) test by labelling bars with different lowercase letter (a, b, and c).

To mimic natural bacterial infection of tomato leaves without causing physical damage in cells, we optimized the dipping method without using the vacuum infiltration. *Pst* DC3000 infection symptoms and bacterial growth were recorded until 7 days post-inoculation (dpi) (Figure 1, Supplementary Figure S1). The growth of *Pst* DC3000 started 4 h post-inoculation (hpi) and dramatically increased at 3 dpi (Figure 1B) although the disease phenotype became more obvious at 3, 5 or 7 dpi.

Mysore et al. [22] have examined the transcriptomic change in tomato upon 0 to 8 hpi of *Pst* and showed that the majority of defence-related transcriptional responses were significantly activated at 8 hpi. In addition, Parker et al. have examined the proteome change of tomato after 4 and 24 h inoculation of *Pst* using isobaric tags for relative and absolute quantitation (iTRAQ) approach and the 24 hpi of *Pst* was considered as the later time point for detecting the ETI responses [18]. In this study, in order to cover early to the later phases of the pathogenesis responses in protein level, 4, 8 and 24 hpi of *Pst* DC3000 were selected for the proteomics analysis. Three different pooled tryptic peptide samples, each combining *Pst* DC3000-inoculated and mock-treated samples from the same time point, were used in the data-dependent acquisition (DDA) method and results were searched from 3 different programs, Mascot, X!tandem and Comet, which were then merged to construct the spectral library. In total, 2174 proteins were identified by DIA analysis across the 3 time points of 4, 8 and 24 hpi and the mock group, at 4, 8, 24 h-post treatment (hpt). Of 1472 proteins being quantified in 3 biological replicates, 114, 147 and 337 proteins had a change in quantity between inoculation and mock at 4, 8, 24 h time points, respectively using the Student’s t-test (Supplementary Tables S1 and S2). Among those, 20, 65 and 189 proteins had significant change in abundance with fold change greater than 1.5 or less than 0.67 at 4, 8 and 24 hpi, respectively, compared to the mock (*p* < 0.05). Comparing to the previous protein quantitation results using iTRAQ approach, even though > 1.2- or < 0.83-fold change with *p*-value < 0.05 was used to identify differentially expressed proteins, there were 128 tomato proteins found to be regulated at 24 hpi of *Pst* DC3000 [18]. In this study, we identified 189 regulated proteins at 24 hpi using the protein fold change >1.5 or <0.67 with *p*-value < 0.05 as the criteria. The higher number of regulated proteins identified in this study as compared to previous iTRAQ study at 24 hpi may possibly due to different experimental design or the ratio compression issue of the use of isobaric tagging proteomics approach.

**Figure 2:**
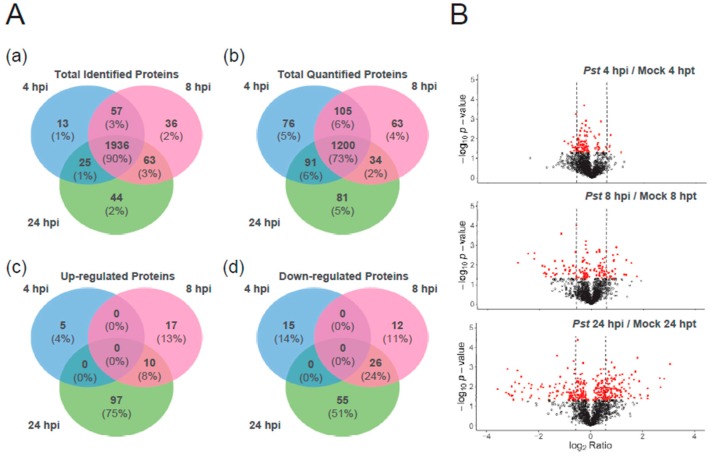
Proteomic change in tomato leaf between time points of *Pst* DC3000 inoculation 4, 8 or 24 hpi compared to each mock: 4, 8 or 24 hpt, respectively. (**A**) Venn diagrams showing unique and shared proteins between 3 time points which are (a) identified across replicates and (b) quantified in all 3 biological replicates or with significant increase, with fold change (c) greater than 1.5 or (d) less than 0.67 in quantity (*p* < 0.05), due to inoculation. (**B**) Volcano plots showing protein abundance ratio of *Pst* DC3000-inoculated over mock group at 4 hpi, 8 hpi and 24 hpi. Following LC-MS analysis and DIA quantification, *t* test-based significance values (log_10_ (*p*-value)) were plotted versus log_2_ (protein quantity ratio for all proteins between infected and mock). Differentially regulated proteins with *p* < 0.05 are plotted in red. Proteins with *p* > 0.05 are plotted in black. The level of protein abundance change with 1.5 or 0.67-fold is marked by a dashed line. The data of quantified proteins in 3 biological replicates were listed in Supplementary Table 1 while the data of each replicate were listed in Supplementary Table 2.

The majority of proteins (~90%) were identified in all 3 time points and only about 1 to 2% of the proteins were identified at one time point (Figure 2A). A similar situation was observed with the quantified proteins, most of which were shared between all the time points. However, there were no proteins with significant up- (>1.5-fold change) or down- (<0.67-fold change) regulation found at all the time points and only ~8% of up-regulated or ~24% of down-regulated proteins were shared between the 8 and 24 h time points. There were also more proteins with significant change, either up- or down-regulated, at 24 hpi than 8 or 4 hpi (Figure 2B), indicating that between 4, 8 and 24 hpi, a different set of proteins were regulated in tomato leaves. More proteins showed a change in quantity at both 8 and 24 hpi, than between 4 and 24 or 4 and 8 hpi.

### 2.2. Functional Classification of the Proteins Regulated by Pst DC3000

#### 2.2.1. Function Categories of Proteins That are Significantly up- or down-Regulated during Pst DC3000 Inoculation

**Figure 3:**
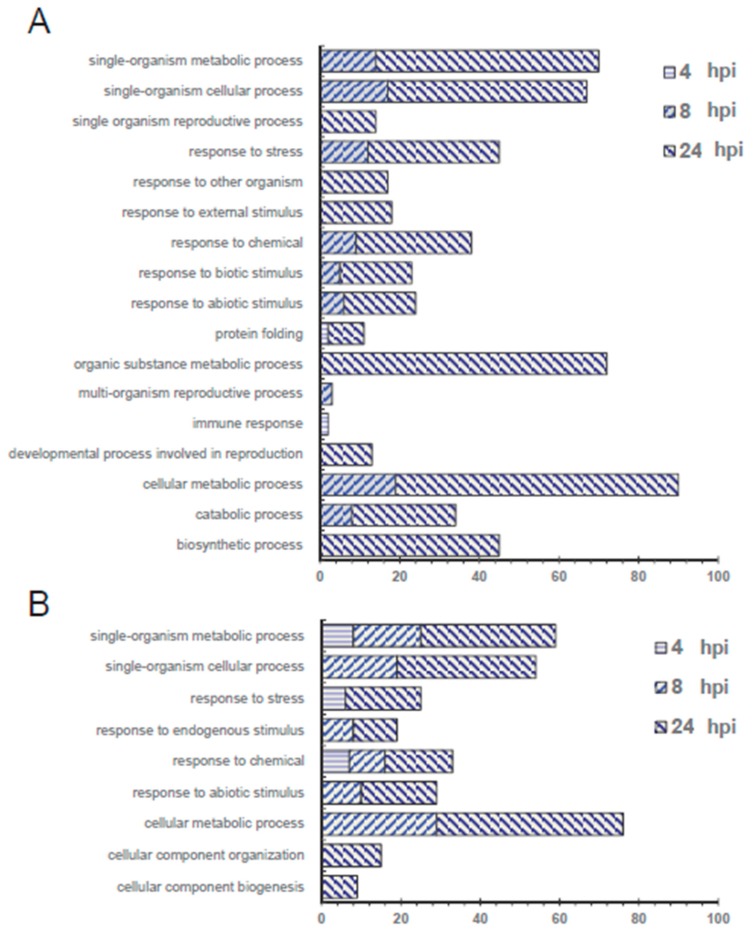
Biological process function analysis for tomato leaf proteins with a (**A**) significant increase or (**B**) decrease in quantity at different infection time points, 4, 8 or 24 hpi compared to the mock group. Only proteins with fold change greater than 1.5 or less than 0.67 (*p* < 0.05) were analysed and used for searching the Arabidopsis homolog proteins by Protein Basic Local Alignment Search Tool (BLASTP) against the Arabidopsis Information Resource (TAIR) database. Gene Ontology (GO) categorization of these Arabidopsis homolog proteins was performed by using the Database for Annotation, Visualization and Integrated Discovery (DAVID) v6.8.

Because there are more thorough gene annotations in the Arabidopsis Information Resource (TAIR) database, using Protein Basic Local Alignment Search Tool (BLASTP), Arabidopsis homolog proteins were identified in TAIR from the tomato proteins that had a significant change between the inoculated and mock group. Regulated proteins with >1.5 or <0.67-fold change were categorized by protein function (Figure 3). More unique function categories were identified for the up-regulated proteins than the down-regulated proteins, including “response to other organism,” “response to external stimulus,” “response to biotic stimulus,” “organic substance metabolic process,” “multi-organism reproductive process,” “immune response” and “developmental process involved in reproduction.” This suggests that the proteins in these categories could be positive regulators in pathogen defence and immune responses. On the other hand, only one category, “response to endogenous stimulus,” was identified in the down-regulated proteins.

#### 2.2.2. Proteins Related to the Key Mechanisms of Pathogenesis

About 70 proteins with significant change in protein abundance upon Pst DC3000 inoculation across 4, 8 and 24 hpi were selected and grouped with their protein function category (Table 1, Table 2 and Table 3). Table 1 summarizes the proteins involved in defence, immune response and ROS (reactive oxygen species)/redox metabolism. Proteins involved in these categories mostly were up-regulated at 8 and/or 24 hpi, except zeaxanthin epoxidase (ZEP; Solyc02g090890) and one peroxisome (Solyc09g072700). Interestingly, none of these were up-regulated at the early time point 4 hpi. Several pathogenesis-responsive proteins with known ROS/redox function were found to be differentially expressed in our results. However, these proteins did not show significant change in quantities in either the Pst-resistant (PtoR) or susceptible (Prf3) tomato genotypes at 4 hpi and 24 hpi in the iTRAQ analysis [18], suggesting our findings could help researchers identify more protein candidates in response to pathogenesis. Table 2 summarizes proteins participating in protein translation, protein folding, degradation and transportation. Most proteins with function in translation and folding were up-regulated at 24 hpi. However, Heat shock proteins 90 (HSP90) and calnexin were the two early up-regulated proteins at 4 hpi. The protein product of Solyc04g080960, pre-pro-cysteine proteinase, also named as RD19A-like and DNA damage-inducible protein 1 (Solyc10g005890) were the ones with down-regulated protein level. DNA damage-inducible protein 1 belongs to a family of shuttle proteins targeting polyubiquitinated substrates for proteasomal degradation. Table 3 summarizes proteins involved in carbohydrate and energy metabolisms, including glycolysis/tricarboxylic acid (TCA) cycle, pentose phosphate pathway and carbon fixation (photosynthesis). Generally speaking, proteins involved in energy-generation, such as glycolysis and the TCA cycle, were up-regulated at 8 and 24 hpi; while proteins involved in photosynthesis and ATP synthesis were all down-regulated. Our data indicate that within the first 24 h of pathogenesis progression in plants, the plant required more energy for defence response and sacrificed the carbon fixation process. Table 4 summarizes proteins involved in oxidation phosphorylation, amino acid, fatty acid and secondary metabolisms like polyamine synthesis and shikimate pathway. Proteins grouped in amino acid metabolisms were up-regulated at 24 hpi and the downstream compounds of these metabolic pathways are involved in the stress response, like polyamine, flavonoids and aromatic amino acid and phytohormones.

### 2.3. Changes Associated with Defence and Immune Regulation

In this study, several proteins involved in the defence of pathogenesis were observed to be regulated mainly at 8 and 24 hpi. The two chitinases, pathogenesis-related protein 4 (PR-4) and chitinase Z15140 and major allergen Pru ar 1 (also known as Pathogenesis-Related Protein-STH 2-like; PR-STH 2-like) were found to be regulated at 8 hpi. Chitinases are enzymes catalysing the hydrolysis of chitin into *N*-acetyl-d-glucosamine monomers therefore this enzyme can damage fungal cell walls and the exoskeleton of arthropods. PR-4 was found to be induced not only by pathogen attack but also induced by ethylene in Arabidopsis [23,24]. Although PR-4 was classified as an endochitinase, its chitinase activity was found to be weak [25]. A more recent study indicated that PR-4 is a bifunctional enzyme with both RNase and DNase activity [26,27], which suggests that PR-4 may be involved in the regulation of HR. It was reported that the transient expression of PR-4b triggers hypersensitive cell death in Arabidopsis and the induction of PR4b is necessary to defend *P. syringae* and *Hyaloperonospora arabidopsidis* (Hpa) infection [28]. For major allergen Pru ar 1, this protein was also named as PR-10a and found to be induced after wounding, elicitor treatment or infection by *Phytophthora infestans* of the plants [29]. It has been shown that the parsley homologs of PR-l0a are highly similar to a partial sequence of a ginseng ribonuclease [30], suggesting the protein encoded by the PR-10a domain could exhibit ribonuclease activity. Moreover, many of these genes have been shown to be controlled at the transcriptional level and to be expressed during the defence response or after wounding [24,31,32]. The function of the PR-10a protein is currently unknown.

At the later time point, 24 hpi, immune-related proteins, including the well-known SA-responsive marker genes/proteins, pathogenesis-related protein 1 (PR-1; Solyc00g174340), Kunitz trypsin inhibitor (KTI; Solyc03g098730) and arginase 2 (ARG2; Solyc01g091170) appeared to be dramatically up-regulated with up to 8-fold increase compared to the mock. PR-1 was observed to be highly up-regulated specifically at 24 hpi with a fold change of ~8.33. PR-1 is triggered by SA, a hormone in the deployment of systemic acquired resistance (SAR). SAR is the defence response occurring at the non-infected site (and thus called “systemic”) to establish a long-term and broad-spectrum resistance throughout the entire plant after local pathogen infection is activated by avirulent pathogens [33]. Activation of the PR-1 gene requires the recruitment of a transcriptional enhanceosome to its promoter for which the SA receptor, Non-Expressor of Pathogenesis-Related Gene 1 (NPR1), is the key regulator [34]. Our previous study demonstrated the function of the PR-1 in the regulation of the immunity, in which PR-1 acts as a precursor for the signalling peptide CAPE1 to trigger defence responses regulated by methyl-jasmonic acid (MeJA) and SA [5]. We also demonstrated that the production of CAPE1 in tomato is regulated by the wounding response and MeJA may further enhance the production of this peptide elicitor [5]. This implies that the plant defence mechanism had started to significantly activate the SAR at 24 hpi. The wound- or JA-dependent responses during the later stage of pathogenesis response may further trigger CAPE1 to induce different sets of the defence responses. On the other hand, the protease KTI, which belongs to the Kunitz-type protease inhibitor (PI) whose serine protease activity specifically inhibits trypsin proteases which cleave polypeptides at the C-terminus of lysine and arginine, was up-regulated ~1.64-fold at 24 hpi of *Pst* DC3000. The PI family are usually induced by JA-mediated response to wounding, pathogens or herbivore attack. The Arabidopsis homolog of Kunitz trypsin inhibitor, ARABIDOPSIS THALIANA KUNITZ TRYPSIN INHIBITOR 1 (AtKTI1), is a functional serine protease inhibitor that antagonizes pathogen-associated programmed cell death [35]. The gene expression of AtKTI1 is increased by 24 hpi of *Pst* DC3000 (especially with the effector avrB) in the microarray database [36] and also induced by H_2_O_2_, wounding, SA and some programmed cell death (PCD)-eliciting toxins from necrotrophic fungal pathogens. Since PCD, a cell-suicide act that needs to be tightly controlled for plant development and pathogen defence, it is speculated that plant induces KTI1 in the infected tissue in order to finely control the fate of plant cells under hemi-biotrophic pathogen inoculation. Previously researchers also found that AtKTI1 in plants can trigger both SA- and JA/ET-dependent defence gene expression, indicating the diverse role of this protein in defence responses including PTI and ETI [35]. In addition, another immune related protein ARG2 was observed to be ~ 6.5-fold expressed when inoculated by *Pst DC3000* only at 24 hpi. ARG2 targets the mitochondria, hydrolyses the first step of arginine degradation to ornithine and urea, also provides the upstream production of proline, histidine and the polyamine biosynthetic pathway [37]. It has been found that the level protein expression and enzyme activity can be induced by wounding, JA treatment and *Pst* DC3000 phytotoxin coronatine in tomato [38]. In our study, ARG2 was severely up-regulated; however, in previously published research, the RNA level was induced as early as 1 hpi and peaked at 8 hpi [38]. This difference could be due to the difference between the different pathogen inoculation methods, as our method could avoid causing wounding compared to the traditional vacuum infiltration in the early stage of the treatment and thus delay the overexpression at the translational level. Besides polyamine, ARG2 may be involved in regulating nitric oxide (NO) accumulation. ARG2 has been shown to be important in the defence against necrotrophic pathogen *Botrytis elliptica* [39]. NO-mediated defence and immunity could be related to SA-JA antagonism as NO induces the accumulation of SA while inhibiting the expression of JA-responsive genes [40]. The late-induction level of ARG2 suggests that the HR-directed PCD by NO signalling could be triggered at 24 hpi.

### 2.4. Changes Associated with the Reactive Oxygen Species (ROS) and Oxidation-Reduction Reactions

ROS, including hydrogen peroxide (H_2_O_2_) and superoxide (O_2_^−^) are involved in the early signalling for defence responses and also has direct toxicity against pathogen function, underlying PTI, ETI and SAR. The activity for mediating rapid accumulation of ROS during the oxidative burst is dependent on two classes of enzymes: NADPH oxidases and class III heme peroxidases [41,42]. NADPH oxidases synthesize the superoxide and peroxidases convert superoxide into a more stable form of ROS, H_2_O_2_. One peroxidase (Solyc04g071900) which belongs to the class III heme peroxidases was observed to be ~1.8-fold up-regulated at 8 hpi, suggesting a diverse ROS-related mechanism could be triggered at 8 hpi. Glutathione S-transferases (GSTs) are known for their function in anti-oxidative reactions to eliminate ROS and lipid hydroperoxides that accumulate in infected tissues thus limiting the excessive spread of HR- associated cell death [43]. Several GST family members have been shown to be PAMP-responsive genes in Arabidopsis [44]. In our study, the major antioxidant enzymes including several GSTs and GST-like proteins and glutathione reductase (GR) were up-regulated at 8 and/or 24 hpi upon *Pst* DC3000 inoculation.

In addition to glutathione and ascorbate, the most abundant antioxidant compounds in plant cells are oxidoreduction-active proteins called redoxins [45]. Thioredoxin (Trx) is a multigenic superfamily of ubiquitous redox proteins with multiple functions. Thioredoxin reductase (Solyc02g082250), one of ROS-detoxifying enzymes, was also up-regulated upon *Pst* DC3000 infection at 8 hpi, confirming that plants also trigger the redox change by thioredoxin reductase during pathogen inoculation.

Although the up-regulation of plant GST proteins as a consequence of bacterium-induced oxidative stress was recognized as above mentioned, there were also some predicted peroxidases, GST proteins and ascorbate peroxidase (Table 1 & Supplementary Table 1) were down-regulated at *Pst* DC3000 at 24 hpi. This is probably due to the effect of SA mediating ROS accumulation in cells by first promoting ROS accumulation (as these ROS are essential in the early defence response) and then inhibiting catalase and cytosolic ascorbate peroxidase, the main H_2_O_2_-detoxifying enzymes, to promote further accumulation of ROS which triggers the ETI response [37,46]. The effectiveness of SA-mediating defence response is also supported by our observation that between 4 and 24 hpi, the colony growth did not show significant change in our experiments (Figure 1B).

### 2.5. Changes Associated with Protein Folding, Transportation and Degradation

#### 2.5.1. Protein Folding: Heat Shock Proteins and Chaperones

Heat shock proteins (HSPs) are a huge protein family participating in plant growth, development and fitness, mainly by helping mature protein folding or degrading mis-folded proteins. HSPs like HSP70, HSP90 and HSP60 belong to molecular chaperone families. Molecular chaperones bind and catalytically unfold misfolded and aggregated proteins as a primary cellular defensive and housekeeping function [47]. Plastidic chaperonin 60 (CPN60) alpha and beta are required for plastid division in Arabidopsis. CPN60 proteins are required to be maintained at a proper level for folding stromal plastid division proteins and are essential for the development of chloroplasts [48]. In our study, CPN60 alpha subunit (Solyc06g075010) was up-regulated by ~2.8-fold, suggesting its important role in bacteria defence response.

The HSP90 family have been shown to be involved in regulating drought, salt and oxidative stress and involved in ATP-dependent assembly of the 26S proteasome [49]. We found that at *Pst* DC3000 4 hpi in tomato, HSP90 (Solyc06g036290) was induced ~2.2-fold compared to the mock. It has been suggested that HSP90 protein could be an important helper to the regulation of the receptor proteins involved in plant immunity, nucleotide-binding site leucine-rich repeat (NB-LRR) type R proteins. This interaction is apparently required for the NB-LRR type R proteins to maintain the protein stability, especially their sensor signal-competent state [50]. HSP90 could physically interact with various R proteins, including RPM1 (RESISTANCE TO PSEUDOMONAS MACULICOLA 1), RPS2 (RESISTANCE TO P. SYRINGAE 2) and RPS4 (RESISTANCE TO PSEUDOMONAS SYRINGAE 4) in Arabidopsis [51,52]. In *Nicotiana benthamiana*, the complex formed by HSP90 and Suppressor of the G2 Allele of *skp1* (SGT1) or Required for Mla12 Resistance 1 (RAR1) which is required for resistance gene *Mla12* or both, is essential for plant survival against tobacco mosaic virus [53,54]. Our data showed that the quick induction of HSP90 at 4 hpi may function in improving the protein stability of NB-LRR, possibly by helping the protein folding, thus enhancing pathogen recognition and signal transduction.

#### 2.5.2. Protein Degradation

The papain-like Cathepsin B-like cysteine protease (CathB), localized in the apoplast region, has proven to be a positive regulator of the HR defence [55]. One predicted gene product of Solyc02g069110, CathB protein, was dramatically increased in protein quantity at 24 hpi with ~3.5-fold. This protease family has endopeptidase activity at the C-terminus of the YVAD substrate and the enzyme activity was restricted predominately to acidic pH [56]. At 8 h of *Pst* DC3000 inoculation in tomato, Solyc04g080960 protein product, a cysteine protease (also known as Response to Dehydration 19A-like proteases; RD19A-like) was down-regulated with 0.64-fold change compared to mock treatment (Table 2). Liu and colleagues recently found that the expression level of Solyc04g080960 was reduced after 24 h of inoculation of the hemi-biotrophic pathogen *Fusarium oxysporum* f. sp. *lycopersici* (FOL) in tomato [57]. On the other hand, RD19 protease activity is required for RPS1-R-dependent immune activation to enhance resistance against the necrotrophic pathogen *Ralstonia solanacearum* by being the interacting protein of *R. solanacearum* type III effector, Pseudomonas outer protein P2 (PopP2), thus initiating the downstream resistance response [58]. In Arabidopsis, AtRD19A also acts as an important protein marker for dehydration stress adaptation and could be highly induced by drought and salt stresses [59]. Taking our quantitative results into consideration, RD19A-like protein may be one of the responsive ends to the danger signals from necrotrophic pathogen attack and abiotic stress like drought or salt stress. Therefore, during the infection of *Pst* DC3000, plants could down-regulate RD19A-like protein in order to be more cost-effective in defence response.

### 2.6. Changes Associated with Phytohormone Synthesis and Fatty Acid Metabolism

In our data, Zeaxanthin epoxidase (ZEP; Solyc02g090890), participating in the biosynthetic pathway upstream of ABA (abscisic acid), was down-regulated at 24 hpi by ~0.61-fold (Table 1). This kind of down-regulation could be the result of the antagonism between ABA and SA [60], since the phytohormone SA is induced by diverse biotrophic/hemi-biotrophic pathogens to activate SAR responses [54].

It has been long believed that there is also antagonism between SA and JA, including the control of each one’s biosynthetic pathway. In our data, several proteins possibly involved upstream of the JA-biosynthetic pathway have been identified, such as fatty acid beta-oxidation multifunctional protein (Absent In Melanoma 1-like; AIM1-like; Solyc12g007170) which showed a ~6.3-fold increase at 24 hpi and lipoxygenase (Solyc01g006560) with ~1.7-fold increase at 24 hpi. The results of 24 h-time point suggest that the JA/ET-mediated defence response could be induced. Our results showed the ET biosynthetic enzyme, 1-aminocyclopropane-1-carboxylate oxidase 1 (ACO; Solyc07g049530) was dramatically up-regulated at the 24 h-time point by ~7-fold. Several S-adenosylmethionine synthase family proteins were also up-regulated by ~2.3-fold at 24 hpi and they are involved in the shikimic acid pathway, which is the upstream of ET and polyamine biosynthesis, suggesting the level of ET should be hugely increased at this late time point.

## 3. Materials and Methods

### 3.1. Plant Materials and Growth Condition

Tomato seeds (*Solanum lycopersicum* cv CL5915, originally provided by AVRDC—The World Vegetable Centre at Tainan, Taiwan) were germinated in soil and grown in a growth chamber for 4–5 weeks. The growth chamber condition was set as 25 °C/22 °C (day/night) temperature, 50%/70% (day/night) humidity and 16 h/8 h (day/night) photoperiod using the light source providing photosynthetic photon flux density (PPFD) 80 µmole/m^2^∙s. Leaflets with similar size from the 3rd to 5th pair of true leaves were collected for the following inoculation experiment.

### 3.2. Pseudomonas Preparation and Inoculation Assays

*Pst* DC3000 from B. N. Kunkel (Washington University, St. Louis, MO, USA) [61] was used as a pathogenic strain on tomato plants. The growth and isolation method were referred to the work published by Desclos-Theveniau et al. [62]. The dipping inoculation method for detached leaf is adapted from previous studies [18,63]. *Pst* DC3000 was grown overnight in liquid King’s B medium [64] with 50 μg/mL rifampicin at 28 °C then resuspended in 10 mM MgSO_4_ and *Pst* DC3000 suspension was adjusted to OD_600_ of 0.02 (~10^7^ cfu/mL). The detached leaves from tomato plants were dipped in *Pst* DC3000 suspension solution with 0.005% Silwet L-77 for 2 min and then placed on water-saturated paper in a petri dish. The dishes were covered and incubated in the growth chamber. The control inoculum as the mock group contained the same components as the bacteria inoculum but without *Pst* DC3000. Disease symptoms and bacterial population were evaluated 0, 4, 8, 24 h and 3, 5, 7 days after inoculation. To estimate the internal bacterial population, leaves were surface sterilized with 70% ethanol, washed twice with sterile distilled water and then homogenized in 10 mM MgSO_4_. The solution was diluted and spotted onto the King’s B agar medium [64] with 50 μg/mL rifampicin. Colonies were counted and reported as means and standard deviations of results for three biological replicates.

### 3.3. Sample Preparation: Protein Extraction and Digestion

Three biological replicates of mock-treated (4, 8, 24 hpt) and *Pst* DC3000 inoculated (4, 8, 24 hpi) leaves were prepared for the proteomics experiments. For each sample, leaves were ground into powder in the chilled mortar and pestle with liquid N_2_ then 0.5 g of powder was collected. The sample was then homogenized with 2.5 mL of ice-cold homogenization buffer (50 mM HEPES-KOH, pH 7.5, 250 mM sucrose, 5% glycerol stock, 10 mM EDTA, pH 8.0, 0.5% Soluble polyvinylpyrrolidone (PVP-10), 3 mM dithiothreitol, 1 mM phenylmethylsulfonyl fluoride and 1× protease inhibitor cocktail) by vortexing for at least 3 min or until completely homogenized. The sample was further incubated in the buffer by Intelli Mixer RM-2L (ELMI Ltd., Riga, Latvia) in the cold room for 30 min. The homogenate was filtered through two layers of miracloth. The volume of filtrate should be relatively close to the initial amount of added homogenization buffer. The supernatant of the filtrate was then collected by centrifugation at 15,000× *g* for 10 min under 4 °C. The protein concentration of each sample was measured by the Bradford assay to quantify the protein amount of total protein.

For each sample, 100 μg of the total protein was precipitated by addition of acetone to 80% with incubation at −20 °C overnight and recovered by centrifugation at 16,000× *g* for 15 min under 4 °C. The reduction and alkylation steps were adapted from the literature as previously described [65]. Re-solubilized proteins were reduced in 50 mM ammonium bicarbonate (ABC) buffer and 8 M urea with 5 mM tris(2-carboxyethyl)phosphine hydrochloride (TCEP) for 1 h at 37 °C and alkylated using 20 mM iodoacetamide for 45 min in the dark at room temperature. Each sample was diluted 4-fold using 50 mM ABC to decrease the urea concentration to less than 2 M then digested with lysyl endopeptidase (LysC; Wako Chemicals, Japan) to a final ratio of 1:50 at room temperature for 3 h. Next the sample was diluted using 50 mM ABC to decrease the urea concentration to ~1 M before digestion with sequencing grade trypsin (Promega, Madison, WI, USA) to a final ratio of 1:50. The proteolysis was continued overnight (14 h) at room temperature and terminated by addition of formic acid to a final concentration of 1% (vol/vol). The digest sample was then desalted using the 50-mg tC18 SepPak cartridge (Waters Corporation, Milford, MA, USA) as described previously [66]. The tryptic peptides from individual sample was dissolved by deionized water containing 2% acetonitrile and 0.1% (v/v) formic acid to the concentration of 500 ng/μL. Three different pooled tryptic peptide samples for DDA analyses in order to construct the DIA spectral library were prepared by combining 4 μg peptides from the same time point of *Pst* DC3000-inoculated and mock-treated sample. For the purpose of retention time calibration, the iRT-standard peptides (Biognosys, Schlieren, Switzerland) were added into the pooled sample and also each individual sample at 1/10 by volume.

### 3.4. Liquid Chromatography-Mass Spectrometry Analysis

The nanoLC-MS/MS was equipped with a self-packed tunnel-frit [67] analytical column (ID 75 μm × 50 cm length) packed with ReproSil-Pur 120A C18-AQ 1.9 μm (Dr. Maisch GmbH, Ammerbuch-Entringen, Germany) at 40 °C on a nanoACQUITY UPLC System (Waters Corporation, Milford, MA, USA) connected to a Q Exactive HF Hybrid Quadrupole-Orbitrap mass spectrometer (Thermo Scientific, Bellefonte, PA, USA). The peptides were separated by a 135-min gradient using the mobile phases including Solvent A (0.1% (*v*/*v*) formic acid) and Solvent B (acetonitrile with 0.1% formic acid). With a flow rate of 250 nL/min, the gradient started with a 40 min equilibration maintained at 2% of B and set as the following segments: 2 to 8% of B in 8 min, 8 to 25% of B in 90 min, then 25% to 48% of B in 5 min, 48 to 80% of B in another 5 min followed by 80% of B wash 10 min and the last equilibrium to 2% B in the last 20 min.

The instrumentation and parameters for DDA and DIA analysis were referred to the previous studies using Q Exactive HF Hybrid Quadrupole-Orbitrap mass spectrometer [68,69]. Two micrograms of the pooled and individual tryptic peptide samples were analysed by DDA and DIA mode, respectively. For DDA analysis, the MS instrument was operated in the positive ion mode and DDA methods for detection of proteome. The instrument was configured to collect high resolution (*R* = 60,000 at *m*/*z* 200 at an automatic gain control target of 3.0 × 10^6^) broadband mass spectra (*m*/*z* 350–1650 Da) with a maximum IT of 20 ms and MS/MS events (*R* = 15,000 at an automatic gain control target of 1.0 × 10^5^) with a dd-MS² IT of 25 ms when a precursor ion charge was 2+, 3+, 4+ and 5+ and an intensity greater than 1.0 × 10^4^, isolation window was set to 1.6 m/z, was detected. The 15 most abundant peptide molecular ions, dynamically determined from the MS1 scan, were selected for MS/MS using a relative higher energy collisional dissociation (HCD) energy of 28% with the dynamic exclusion was 35 s. For DIA analysis, MS/MS proteome profiling, was analysed by the same LC-MS/MS system. The instrument was operated in the positive ion mode and configured to collect high resolution (*R* = 120,000 at *m*/*z* 200 at an automatic gain control target of 3.0 × 10^6^) broadband mass spectra (m/z 350–1650 Da) with a maximum IT of 60 ms and MS/MS events (*R* = 30,000 at an automatic gain control target of 3.0 × 10^6^) with an auto MS² IT, isolation window was set to 52.0 *m*/*z*, fixed first mass was set to 200 *m*/*z*. The 25 segments were selected for MS/MS using a relative higher energy collisional dissociation (HCD) energy of 28%. The acquisition window covered a mass range from 350 to 1650 *m*/*z* through 25 consecutive isolation windows.

### 3.5. Data Analysis for LC-MS

With the DDA data files, the Mascot (ver. 2.3, http://www.matrixscience.com/), X!Tandem (ver. 2013.06.15.1) [70] and Comet (ver. 2017.01 rev.1) [71] were used to do a protein database search against a combined database of ITAG (ver. 3.1, https://solgenomics.net/organism/Solanum lycopersicum/genome; 34881 entries reverse sequence generate as the decoy database) and the iRT standard peptides and BSA (SwissProt Accession: P02769) sequence. Search parameters were set as follows: MS tolerance, 20 ppm, allow precursor monoisotopic mass isotope error; number of trypsin missed cleavage: 2; Fragment Mass tolerance, 0.2 Da; enzyme, trypsin; static modifications, carbamidomethyl (Cys, + 57.021 Da); dynamic modifications, oxidation (+15.995 Da) of methionine. Next the software on the Trans-Proteomic Pipeline (TPP, ver. 5.1) [72] was used to combine the search result from different search engines and different repeats; there were a total 116,422 peptide-spectrum matches. In the constructed library, there were a total 67,536 transitions, 9343 peptides and 3070 proteins.

OpenMS (ver. 2.2.0) [73] was utilized for decoyed spectral library construction. We employed the OpenSWATH (ver. 0.1.2) [74] to search the DIA files against the spectral library we constructed. The retention time alignment used the information of iRT transitions. In addition to the chromatogram alignment, the spike-in iRT peptide standards were also used for the quality control of the DDA and DIA analyses. In all DDA and DIA analyses across the sequence of the instrument in the study, the coefficient of variation (CV) of iRT peptide retention time should be less than 3% and the CV of iRT peptide peak intensity should be less than 20%. Search parameters were set as follows: peptide false-discovery rate (FDR), 0.05; protein FDR, 0.01; alignment method, LocalMST; re-alignment method, lowest; retention time (RT) difference, 60; alignment score, 0.05. The ratios of protein quantitation between the *Pst* DC3000-inoculated and mock-treated sample in each replicate were normalized by the most-likely ration normalization principle as previously applied in the DIA study [75].

### 3.6. Quantitation Data Analysis

Only proteins detected and quantified in all runs (3 biological replicates) were included in the data set. To perform a significance test, the students’ t-test was calculated. Any protein with differential abundance with a *p*-value of less than 0.05 and fold change greater than 1.5 or less than 0.67 was defined as being “significantly” regulated in protein quantity. Functional annotations of the quantified tomato proteins were obtained via PANTHERN (ver. 13.1) [76,77]. To show the functional distribution of the regulated proteins, the up- and down-regulated protein sequences were searched against the *Arabidopsis thaliana* TAIR10 database (http://arabidopsis.org) using BLASTP with E-value < 1.0 × 10^−5^ (https://blast.ncbi.nlm.nih.gov/Blast.cgi) first and the matched Arabidopsis homolog proteins were categorized by the GO biological function level 2 using DAVID v6.8 (https://david.ncifcrf.gov/) [78,79].

## 4. Conclusions

This study demonstrated a successful example of using the DIA approach for a time course analysis of plant pathogenesis proteomics. A total of ~2200 proteins were identified and quantified from the tomato subjected to different treatments and 90% of the totally identified proteins were commonly observed across all the treatments. This study indicates different sets of proteins are regulated from the early to the later stage of the *Pst* DC3000 infection. We showed that no defence-related protein was observed to be up-regulated but the chaperone proteins for helping the activity of R proteins was induced at 4 hpi of *Pst* DC3000. Several major defence and immune-related proteins were found to be up-regulated at 8 and 24 hpi. One of the peroxidase proteins related to the production of H_2_O_2_ was up-regulated at 8 hpi. We have shown that plants do not only express proteins for accumulating H_2_O_2_ but also detoxification proteins to avoid the over-accumulation of ROS. The proteins involved in the later stage of the pathogenesis which are related to the HR and PCD were up-regulated at 24 hpi. We also discovered that the proteins involved in the biosynthesis of JA and ET were induced at 24 hpi, indicating ET/JA may be induced in the later pathogenesis response. More time points and treatments can be further analysed and compared with the current DIA datasets based on the library established in this study. The number of proteins identified and quantified with the use of the current DIA approach can also be increased when a more comprehensive spectra library for the tomato proteome is established.

## Figures and Tables

**Table 1:** Proteins involved in defence, immunity and ROS/redox mechanism with significant change in abundance at 4, 8, 24, hpi of *Pst* DC3000.

	Protein Description	Gene Accession		4 hpi			8 hpi			24 hpi	
				log_2_ Ratio ^a^	*p*-Value ^b^		log_2_ Ratio ^a^	*p*-Value ^b^		log_2_ Ratio ^a^	*p*-Value ^b^
**Defence**											
	Pathogenesis-related protein 4 (PR-4)	Solyc01g097240		−0.183	0.496		0.885	0.049		NQ	NQ
	PIN-I protein (PR-6)	Solyc09g083440		−0.023	0.971		−1.163	0.052		1.484	0.004
	Wound-induced proteinase inhibitor 1	Solyc09g084465		NQ	NQ		NQ	NQ		1.183	0.019
	Major allergen Pru ar 1 (PR-STH 2)	Solyc09g090970		0.239	0.038		0.827	0.003		1.106	0.036
	Biotin-binding protein	Solyc09g065540		0.067	0.695		0.093	0.794		0.615	0.048
	Chitinase Z15140	Solyc10g055810		−0.195	0.487		0.869	0.006		0.101	0.907
	Beta-1,3-glucanase (PR-2)	Solyc01g008620		0.123	0.669		0.291	0.290		1.111	0.008
**Immune Regulation**											
	Pathogenesis-related protein 1 (PR-1)	Solyc00g174340		−0.182	0.153		0.949	0.052		3.059	0.001
	1-aminocyclopropane-1-carboxylate oxidase 1	Solyc07g049530		0.132	0.705		NQ	NQ		2.831	0.004
	Arginase 2 (ARG2)	Solyc01g091170		0.407	0.356		0.289	0.466		2.691	0.011
	Cathepsin D Inhibitor	Solyc03g098780		NQ	NQ		NQ	NQ		1.947	0.040
	Activator of 90 kDa heat shock ATPase	Solyc10g078930		0.204	0.442		0.544	0.065		0.936	0.005
	FKBP-like peptidyl-prolyl cis-trans isomerase family protein	Solyc09g008650		0.084	0.827		0.851	0.006		0.925	0.035
	Kunitz trypsin inhibitor	Solyc03g098730		−0.355	0.187		NQ	NQ		0.714	0.046
	Zeaxanthin epoxidase (ZEP)	Solyc02g090890		0.112	0.353		−0.799	0.067		−0.711	0.047
**ROS/Redox**											
	Glutathione S-transferase/peroxidase	Solyc07g056480		0.041	0.814		NQ	NQ		1.870	0.005
	Peroxidase	Solyc09g072700		−0.345	0.089		−0.500	0.336		−0.814	0.029
	Thioredoxin reductase	Solyc02g082250		0.064	0.930		0.813	0.022		NQ	NQ
	Peroxidase	Solyc04g071900		NQ	NQ		0.906	0.016		NQ	NQ
	Glutathione Reductase (GR)	Solyc09g091840		0.476	0.086		0.368	0.290		0.902	0.010
	Glutathione S-transferase-like protein	Solyc09g011570		0.158	0.430		0.765	0.038		0.812	0.001
	Glutathione S-transferase	Solyc06g009040		0.201	0.401		0.865	0.049		0.429	0.210

^a^ The average log_2_ ratio of protein quantity representing (inoculated/mock) from 3 biological replicates. ^b^ *p*-value calculated from Student t-test. Color-coded: red, significant quantity change greater than 0.58 of log_2_ ratio; blue, significant quantity change less than −0.58 of log_2_ ratio; grey, no significant change between the inoculated and mock group (*p* ≥ 0.05); white, non-quantifiable (NQ).

**Table 2:** Proteins involved in translation, protein folding, degradation and transportation with significant change in abundance at 4, 8, 24, hpi of *Pst* DC3000.

	Protein Description	Gene Accession		4 hpi			8 hpi			24 hpi	
				log_2_ Ratio ^a^	*p*-Value ^b^		log_2_ Ratio ^a^	*p*-Value ^b^		log_2_ Ratio ^a^	*p*-Value ^b^
**Protein Translation, Folding, Degradation and Transportation**											
	Importin subunit alpha	Solyc01g060470		−0.122	0.473		0.185	0.449		1.138	0.016
	Protein transport protein sec23, putative	Solyc05g053830		−0.023	0.894		0.262	0.271		1.012	0.016
	Golgin candidate 6	Solyc08g081410		−0.087	0.655		−0.095	0.612		0.680	0.040
	Cathepsin B-like cysteine protease	Solyc02g069110		NQ	NQ		NQ	NQ		1.802	0.000
	T-complex protein 1 subunit beta	Solyc11g069000		−0.487	0.460		0.101	0.597		1.352	0.017
	26S proteasome non-ATPase regulatory subunit 3	Solyc01g111700		0.116	0.811		0.300	0.034		0.960	0.013
	DNA damage-inducible protein 1	Solyc10g005890		−0.612	0.038		−0.417	0.298		−0.021	0.862
	Pre-pro-cysteine proteinase	Solyc04g080960		0.313	0.193		−0.634	0.025		−0.388	0.316
	Chaperonin 60 alpha subunit	Solyc06g075010		−0.128	0.579		−0.080	0.607		1.506	0.012
	Heat shock 70 kDa protein, putative	Solyc07g043560		0.233	0.194		0.251	0.298		1.223	0.002
	Peptidyl-prolyl cis-trans isomerase	Solyc06g076970		0.211	0.697		0.759	0.500		1.212	0.033
	Heat shock protein 90	Solyc06g036290		1.140	0.050		1.475	0.109		0.841	0.050
	Calnexin	Solyc03g118040		0.672	0.028		0.256	0.576		−0.067	0.881

^a^ The average log_2_ ratio of protein quantity representing (inoculated/mock) from 3 biological replicates. ^b^ *p*-value calculated from Student t-test. Color-coded: red, significant quantity change greater than 0.58 of log_2_ ratio; blue, significant quantity change less than −0.58 of log_2_ ratio; grey, no significant change between the inoculated and mock group (*p* ≥ 0.05); white, non-quantifiable (NQ).

**Table 3:** Proteins involved in carbohydrate and energy metabolisms with significant change in abundance at 4, 8, 24, hpi of *Pst* DC3000.

	Protein Description	Gene Accession		4 hpi			8 hpi			24 hpi	
				log_2_ Ratio ^a^	*p*-Value ^b^		log_2_ Ratio ^a^	*p*-Value ^b^		log_2_ Ratio ^a^	*p*-Value ^b^
**Metabolism-Primary-Carbohydrate Metabolisms-Glycolysis & TCA**											
	Glucose-6-phosphate 1-dehydrogenase	Solyc02g093830		0.289	0.111		0.752	0.146		0.955	0.013
	Pyruvate kinase family protein	Solyc10g083720		−0.104	0.326		0.316	0.208		0.915	0.001
	Glyceraldehyde 3-phosphate dehydrogenase	Solyc05g014470		0.219	0.501		0.155	0.400		0.773	0.022
	Glyceraldehyde-3-phosphate dehydrogenase	Solyc04g009030		0.258	0.027		0.196	0.208		0.663	0.004
	2-oxoglutarate dehydrogenase E1 component family protein	Solyc05g054640		−0.193	0.360		0.490	0.007		0.654	0.033
	Fructokinase 2	Solyc06g073190		0.030	0.805		0.254	0.149		0.624	0.019
**Metabolism-Primary-Carbohydrate metabolism-PPP**											
	6-phosphogluconate dehydrogenase, decarboxylating	Solyc04g005160		−0.372	0.392		0.919	0.016		1.221	0.006
	Transaldolase	Solyc00g006800		−0.183	0.302		0.159	0.641		0.604	0.039
**Metabolism-Primary-Carbon fixation**											
	Photosystem II oxygen-evolving complex protein 3	Solyc02g079950		−0.739	0.057		−0.694	0.028		−0.250	0.157
	Chlororespiratory reduction31	Solyc08g082400		−0.529	0.136		−1.402	0.033		−1.397	0.042
	ATP-dependent zinc metalloprotease FTSH protein	Solyc07g055320		0.118	0.646		−1.340	0.029		−2.270	0.008
	Protein CURVATURE THYLAKOID 1A, chloroplastic	Solyc10g011770		−0.072	0.814		−1.558	0.040		−2.373	0.007
	Cytochrome b6-f complex iron-sulphur subunit	Solyc12g005630		−0.087	0.801		−1.758	0.039		−2.812	0.002
	Chlorophyll a-b binding protein, chloroplastic	Solyc07g063600		−0.353	0.601		−1.891	0.011		−2.993	0.047
	Photosystem I reaction centre subunit III	Solyc02g069450		0.220	0.625		−1.496	0.079		−3.137	0.026
	Chlorophyll a/b-binding protein	Solyc03g005760		−0.540	0.493		−1.788	0.024		−3.159	0.024
**Metabolism-Primary-Carbohydrate Metabolism-Others**											
	Sucrose synthase	Solyc07g042550		0.250	0.297		1.258	0.031		1.542	0.031
	Beta-fructofuranosidase	Solyc04g081440		0.261	0.367		NQ	NQ		0.818	0.005
	xyloglucan endotransglucosylase-hydrolase 7	Solyc02g091920		0.066	0.888		0.590	0.022		0.573	0.323
	Starch synthase, chloroplastic/amyloplastic	Solyc03g083095		−0.134	0.339		−0.601	0.013		−0.231	0.588

^a^ The average log_2_ ratio of protein quantity representing (inoculated/mock) from 3 biological replicates. ^b^ *p*-value calculated from Student t-test. Color-coded: red, significant quantity change greater than 0.58 of log_2_ ratio; blue, significant quantity change less than −0.58 of log_2_ ratio; grey, no significant change between the inoculated and mock group (*p* ≥ 0.05); white, non-quantifiable (NQ).

**Table 4:** Protein involved in other primary and secondary metabolisms with significant change in abundance at 4, 8, 24, hpi of *Pst* DC3000.

	Protein Description	Gene Accession		4 hpi			8 hpi			24 hpi	
				log_2_ Ratio ^a^	*p*-Value ^b^		log_2_ Ratio ^a^	*p*-Value ^b^		log_2_ Ratio ^a^	*p*-Value ^b^
**Metabolism-Primary-Oxidative phosphorylation**											
	ATP synthase subunit alpha, chloroplastic	Solyc06g072540		−0.244	0.124		−0.708	0.011		−0.879	0.001
	ATP synthase delta-subunit protein	Solyc12g056830		−0.546	0.011		−1.014	0.018		−1.061	0.020
**Metabolism-Primary-Amino Acid & Polyamine Metabolism**											
	S-adenosylmethionine synthase 2 (SAM2)	Solyc12g099000		0.045	0.924		−0.345	0.564		1.194	0.006
	Chorismate synthase 1 precursor	Solyc04g049350		0.151	0.259		0.335	0.072		0.830	0.030
	S-adenosylmethionine synthase 1 (SAM1)	Solyc01g101060		0.224	0.091		0.190	0.034		0.681	0.012
	S-adenosylmethionine synthase	Solyc10g083970		NQ	NQ		NQ	NQ		0.630	0.007
	Methylthioribose kinase	Solyc01g107550		0.125	0.156		0.333	0.169		0.626	0.005
	Dehydroquinate dehydratase/shikimate: NADP oxidoreductase	Solyc01g067750		−0.191	0.086		0.090	0.755		0.598	0.014
	Ornithine decarboxylase	Solyc04g082030		0.153	0.211		0.728	0.293		1.673	0.037
	Spermidine synthase	Solyc05g005710		−0.494	0.048		−0.020	0.955		1.007	0.014
**Metabolism Primary-Fatty Acid/Lipids**											
	Fatty acid beta-oxidation multifunctional protein	Solyc12g007170		0.340	0.573		0.663	0.204		2.662	0.004
	Acetoacetyl-CoA thiolase	Solyc07g045350		0.210	0.020		0.849	0.050		2.195	0.030
	12-oxophytodienoate reductase 3	Solyc07g007870		−0.045	0.860		0.347	0.198		1.468	0.023
	4-coumarate-CoA ligase	Solyc03g117870		0.004	0.987		0.382	0.233		1.282	0.005
	ATP-citrate synthase, putative	Solyc05g005160		0.091	0.649		0.587	0.045		0.998	0.117
	Lipoxygenase	Solyc01g006560		−0.437	0.184		NQ	NQ		0.767	0.038
**Metabolism-Secondary**											
	5-enolpyruvylshikimate-3-phosphate synthase	Solyc01g091190		0.166	0.744		0.351	0.242		1.442	0.043

^a^ The average log_2_ ratio of protein quantity representing (inoculated/mock) from 3 biological replicates. ^b^ *p*-value calculated from Student t-test. Color-coded: red, significant quantity change greater than 0.58 of log_2_ ratio; blue, significant quantity change less than −0.58 of log_2_ ratio; grey, no significant change between the inoculated and mock group (*p* ≥ 0.05); white, non-quantifiable (NQ).

## Supplementary Materials

Supplementary materials can be found at http://www.mdpi.com/1422-0067/20/4/863/s1. Supplemental Figure S1, disease phenotypes and population dynamics of *Pseudomonas syringae* pv. *tomato* on the tomato leaves at 24 hpi, 3 dip and 7 dpi. Supplemental Table 1, list of quantified proteins with significant change in abundance at 4 hpi, 8 hpi or 24 hpi compared to mock. Supplemental Table 2, full list of quantified proteins and peptides in three biological replicates of the *Pst* DC3000-inoculated (4 hpi, 8 hpi, 24 hpi) and mock-treated (4 hpt, 8 hpt and 24 hpt) experiments. All the mass spectrometry raw data files were deposited to the ProteomeXchange Consortium via the PRIDE [80] partner repository with the dataset identifier PXD012226.

## Abbreviations

DIA	data-independent acquisition
DDA	data-dependent acquisition
PAMP	pathogen-associated molecular pattern
MAMP	microbial associated molecular pattern
DAMP	damaged-associated molecular pattern
ETS	effector-triggered susceptibility
PTI	PAMP-triggered immunity
ETI	effector-triggered immunity
HR	hypersensitive response
PCD	programmed cell death
SA	salicylic acid
JA	jasmonic acid
ABA	abscisic acid
ET	ethylene
*Pst* DC3000	*Pseudomonas syringae* pv. *tomato* DC3000
SAR	systemic acquired resistance
