# Supplementary material for: Application of Data-Independent Acquisition Approach to Study the Proteome Change from Early to Later Phases of Tomato Pathogenesis Responses
Source: Int J Mol Sci. 2019 Feb 17;20(4):863. doi: 10.3390/ijms20040863 (PMC6413104; doi:10.3390/ijms20040863)
Supplement: Supplementary file 1 [file ijms-20-00863-s001.zip › Supplementary data/Figure_S1_rev 190208.pdf]

Supplementary Figure S1

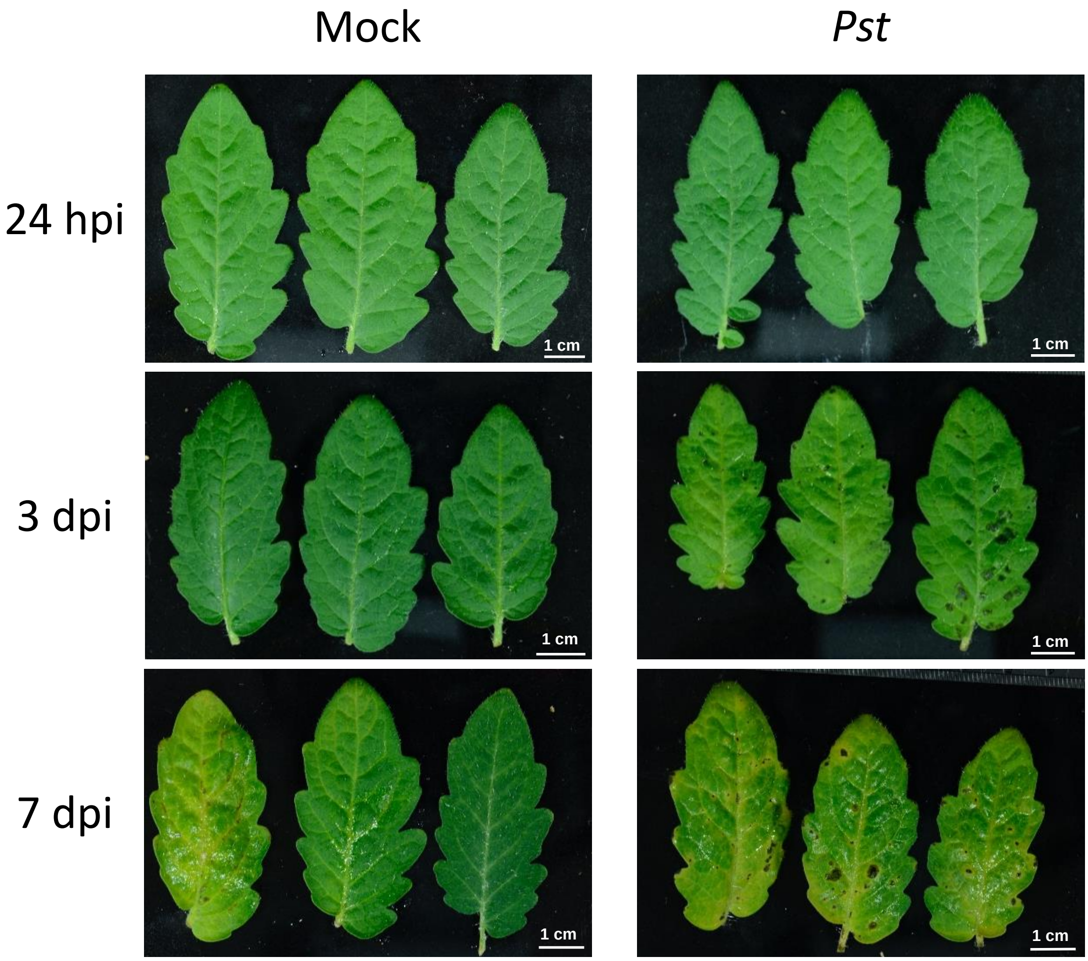

Figure S1. Disease phenotypes and population dynamics of *Pseudomonas syringae* pv. *tomato* on the tomato leaves at 24 hpi, 3 dip and 7 dpi. The leaf color became yellow-green only at 7 dpi sample possibly due to the senescence which could be observed in both mock and *Pst*-inoculated group.
